# Extraction Optimization, Preliminary Characterization and Bioactivities *in Vitro* of *Ligularia hodgsonii* Polysaccharides

**DOI:** 10.3390/ijms17050788

**Published:** 2016-05-21

**Authors:** Xueping Song, Jun Tang

**Affiliations:** Key Laboratory of Combinatorial Biosynthesis and Drug Discovery (Wuhan University), Ministry of Education, and Wuhan University School of Pharmaceutical Sciences, Wuhan 430071, China; XuepingSong@whu.edu.cn

**Keywords:** *Ligularia hodgsonii* Hook., polysaccharides, extraction optimization, characterization, antioxidant activity, anti-hyperglycemic activity

## Abstract

The optimization extraction, preliminary characterization and bioactivities of *Ligularia hodgsonii* polysaccharides were investigated. Based on single-factor experiments and orthogonal array test, the optimum extraction conditions were obtained as follows: extraction time 3 h, temperature 85 °C, water/raw material ratio 36. Further Sevag deproteinization and dialysis yielded the dialyzed *Ligularia hodgsonii* polysaccharides (DLHP, 19.2 ± 1.4 mg/g crude herb). Compositional analysis, size-exclusion chromatography connected with multi-angle laser light-scattering and refractive index (SEC-MALLS-RI), Fourier transform infrared (FT-IR) and ^1^H nuclear magnetic resonance (NMR) spectroscopy were employed for characterization of the polysaccharides. DLHP was found to have a major component with a weight-average molecular weight of 1.17 × 10^5^ Da, mainly comprising of glucose, galactose, arabinose, mannose, rhamnose, glucuronic acid and galacturonic acid. By *in vitro* antioxidant activity assays, DLHP presented remarkable scavenging capacities towards 1,1-diphenyl-2-picrylhydrazyl (DPPH), 2,2′-azinobis (3-ethylbenzothiazoline-6-sulfonic acid) (ABTS) and hydroxyl radicals, and ferrous ions chelating ability. Moreover, it exhibited appreciable anti-hyperglycemic activity as demonstrated by differential inhibition of α-glucosidase and α-amylase. The results indicated that DLHP could potentially be a resource for antioxidant and hypoglycemic agents.

## 1. Introduction

The roots and rhizomes of *Ligularia hodgsonii* Hook. (LH), a medicinal plant belonging to the family Asteraceae, have been widely used for the treatment of cough, excessive phlegm, and inflammation in the Chinese herbal medicines [1]. Recent studies have demonstrated that LH contains essential oils, polysaccharides, phenolic acids, terpenoids and pyrrolizidine alkaloids (PAs) that could contribute to many biological functions of the herb [1,2,3].

It has been reported that excessively high level of free radicals or reactive oxygen species (ROS) caused the oxidative stress, which induced many health disorders such as liver damage, diabetes, and tumor progression [4,5]. Some of the insults (e.g., diabetes and hyperglycemia) might in turn increase the production of ROS [6]. Although the endogenous antioxidant defense could modulate the levels of ROS and avert the harmful effects, it might be difficult to completely abolish the damage even using the conventional antioxidants. Therefore, new, efficient and safe antioxidant agents have been riveting the attention of many researchers, for which a number of naturally-occurring polysaccharides were demonstrated to possess antioxidant, hypoglycemic and hepatoprotective activities [7,8,9]. Some studies also suggested that the antioxidant potential of polysaccharides was closely correlated with other beneficial effects such as anti-diabetes [10]. On the other hand, PAs found in LH are a group of hepatotoxic otonecine-type PAs with clivorine as a representative [11]. These substances may lead to the deactivation of cellular antioxidant enzymes and resultant oxidative stress [12]. Both otonecine-type PAs and polysaccharides are water-soluble and can be co-administered orally in water extract of LH in Chinese medical practice. Previous studies including ours suggested that the LH polysaccharides might have some antioxidant properties and reverse the PA-induced toxicity *in vivo* [1,2,13]. However, despite of the potentially medicinal values, there is still limited literature on the extraction, characterization and bioactive evaluation of the polysaccharides from LH.

In this study, the hot water extraction was performed to separate the LH polysaccharides. The extraction optimization was conducted by single-factor experiments and orthogonal array test. The crude polysaccharides were further purified by Sevag deproteinization and dialysis. Size-exclusion chromatography connected with multi-angle laser light-scattering and refractive index (SEC-MALLS-RI), Fourier transform infrared (FT-IR) and ^1^H nuclear magnetic resonance (^1^H NMR) were applied to analyze and characterize the structures of the polysaccharides. Moreover, *in vitro* bioactivities of the LH polysaccharides, including both antioxidant and anti-hyperglycemic activities, were evaluated.

## 2. Results and Discussion

### 2.1. Optimization Extraction of the Ligularia hodgsonii Hook. (LH) Polysaccharides

**Figure 1:**
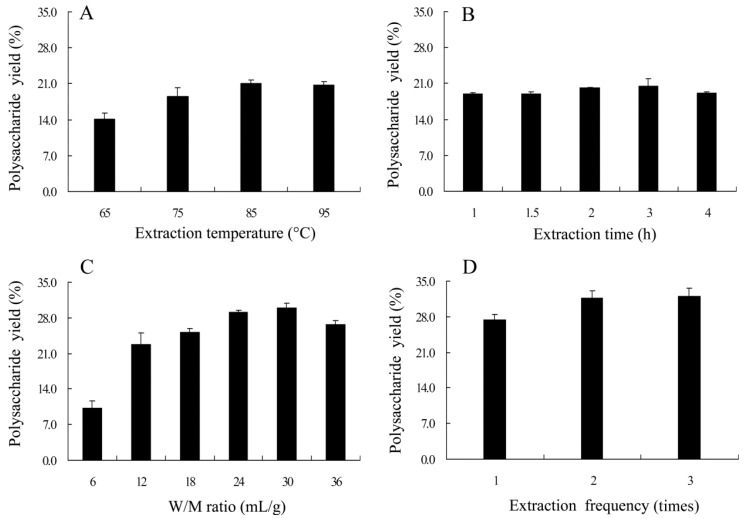
Effects of extraction temperature (**A**); extraction time (**B**); water volume to raw material weight (W/M ratio) (**C**); and extraction frequency (**D**) on the polysaccharide yield.

The effects of different factors on the extraction efficiency of the LH polysaccharides (LHP) were firstly examined using a single-factor test. As observed in Figure 1A, the polysaccharide yield increased rapidly from 14.0% to 21.1% as the extraction temperature heated from 65 to 85 °C, indicating that the high temperature facilitated polysaccharides diffusion to the solvent. However, a slight decline happened at 95 °C in that the high temperature might lead to the partial degradation of the polysaccharides. In Figure 1B, the polysaccharide yield also increased when the extraction time varied from 1 to 3 h, and peaked at 3 h. However, the yield declined slightly to 19.2% at 4 h, which might be owing to gelatinization as the time was prolonged [14]. Figure 1C showed the effects of the ratio of water volume to raw material weight (W/M ratio) on the extraction efficiency. The polysaccharide yield rapidly increased till the ratio was 30:1 (mL/g). Figure 1D indicated that the yield was also influenced by extracting frequency. Extracting twice may be sufficient for the maximum extraction. Even after all of these analyses were conducted, there are still some uncertainties, such as the exact condition for whole extraction processing and the interference of impurities especially toxic PAs to the quality of extracts. Accordingly, an orthogonal design *L*_9_ (3^4^) was performed to further optimize the water extraction condition, in which four main factors (extraction temperature, time, frequency and W/M ratio) and three levels for each factor were selected depending on the above results. As presented in Table 1, the polysaccharide samples were obtained from nine experimental points and determined on the contents of four attributes (the polysaccharide yield, protein, total polyphenols and clivorine) according to the above-mentioned methods. The results showed that run five (A_2_D_1_C_3_B_2_) conducted at extraction temperature 85 °C, extraction time 3 h, and W/M ratio 36 (mL/g) had the highest *Z*-score at 6.35 ± 0.76. In a view of *R* values, the ranking order of all four factors on the polysaccharide yield was A (temperature) > D (frequency) > C (W/M ratio) > B (time). Further variance analysis using factor B (minimum error) as deviation showed that only factor A had significant influence on the extraction efficiency (*p* < 0.05). Because the presence of impurities such as PAs and phenolics may influence the quality and bioactive evaluation of the polysaccharides, the minimum amount of clivorine and total polyphenols in extracts is preferred. As such, the A_2_B_2_C_3_D_1_ combination would be the best procedure (Table 1).

The optimum conditions were further verified by repeating the extraction procedure for three times. The contents of four attributes were also determined; the results showed that the polysaccharide yield was 28.1%, protein content 2.25%, total polyphenol content 1.68%, and clivorine content 8.19 × 10^−5^% on average, respectively (Table 2). A mean *Z*-score of 6.46 ± 0.12 obtained was in good agreement with the predicted value 6.35. This result proved that the optimal conditions were valid for the extraction.

The LHP obtained as above was further purified by Sevag deproteinization and dialysis. The resultant dialyzed LHP (DLHP) was yielded in a ratio of 4.47% ± 0.33% from LHP, or 1.92% ± 0.14% from crude herb. Similarly, the contents of three main impurities (proteins, total polyphenols and clivorine) in DLHP were measured, which were all markedly lower than those of LHP (Table 2).

### 2.2. Preliminary Characterization of the LH Polysaccharides

The LH polysaccharides were subject to further chemical analysis, FT-IR and NMR spectroscopy. The color responses of I_2_-KI test for both LHP and DLHP were negative, while those of Molish reaction positive, indicating that the polysaccharides belonged to carbohydrate material without starch contamination. The contents of total carbohydrate and uronic acids in DLHP were 86.9% ± 0.4% and 5.03% ± 0.08%, respectively, both significantly higher than those of LHP (Table 2). However, there was no sulfate detected in both DLHP and LHP, which was supported by the absence of asymmetrical S=O stretching vibration (around 1230 cm^−1^) and symmetrical C–O–S stretching vibration (around 811 cm^−1^) [9] in FT-IR spectra.

**Figure 2:**
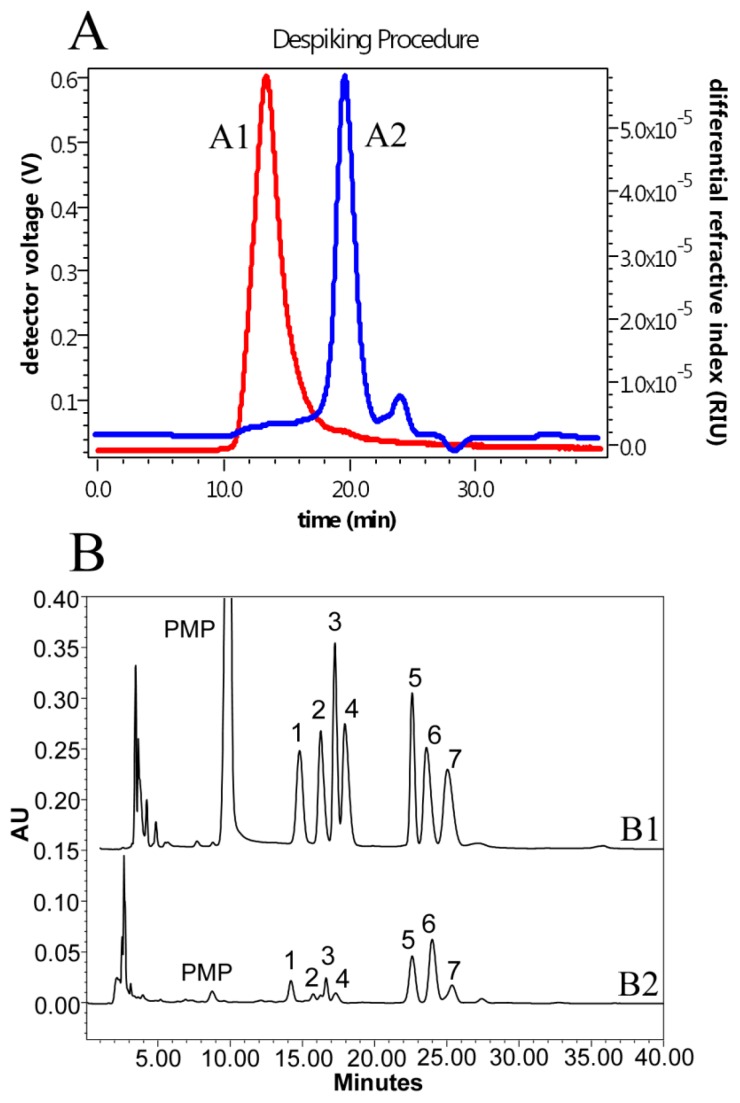
(**A**) Size-exclusion chromatography (SEC) chromatograms of dialyzed *Ligularia hodgsonii* polysaccharides (DLHP) detected by SEC-MALLS-RI at 25 °C. A1 (red line) and A2 (blue line) represent the signals from light scattering (LS) at 90° and differential refractive index (dRI), respectively; and (**B**) HPLC profiles of 1-phenyl-3-methyl-5-pyrazolone (PMP) derivatives of seven monosaccharide standards (B1) and DLHP (B2): 1, Mannose; 2, Galacturonic acid; 3, Rhamnose; 4, Glucuronic acid; 5, Glucose; 6, Galactose; and 7, Arabinose.

SEC-MALLS-RI analyses revealed DLHP containing a major component with *M*_W_, *M*_W_/*M*_n_ and mass fraction of 1.17 × 10^5^ Da, 1.42 and 91.6%, respectively (Table 2). This component showed a single and symmetrically sharp peak, suggesting that it was a homogeneous polysaccharide (Figure 2A). LHP also showed a major component in SEC profile, which *M*_W_ (0.11 × 10^5^ Da) was yet much smaller than that of DLHP. The monosaccharide composition analysis indicated that both DLHP and LHP were composed of mannose, galacturonic acid, rhamnose, glucuronic acid, glucose, galactose, and arabinose, in a relative molar ratio of 0.171:0.064:0.137:0.087:0.521:0.515:0.178 for DLHP (Figure 2B); and 0.073:0.013:0.058:0.120:1.122:0.263:0.298 for LHP. Glucose, galactose and arabinose represented 72.6% and 86.5% of the total monosaccharide detected in DLHP and LHP, respectively, while this percentage of two uronic acids was markedly higher in DLHP (9.07% ± 0.71%) than in LHP (6.82% ± 0.31%).

**Figure 3:**
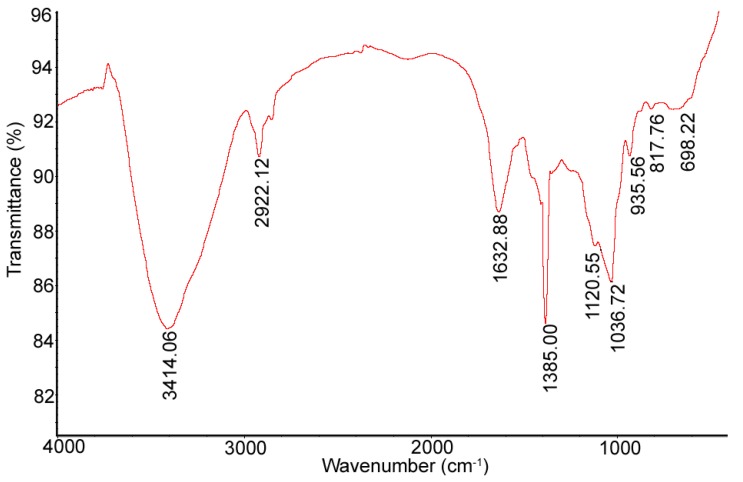
Fourier Transform Infrared (FT-IR) spectrum of DLHP.

**Figure 4:**
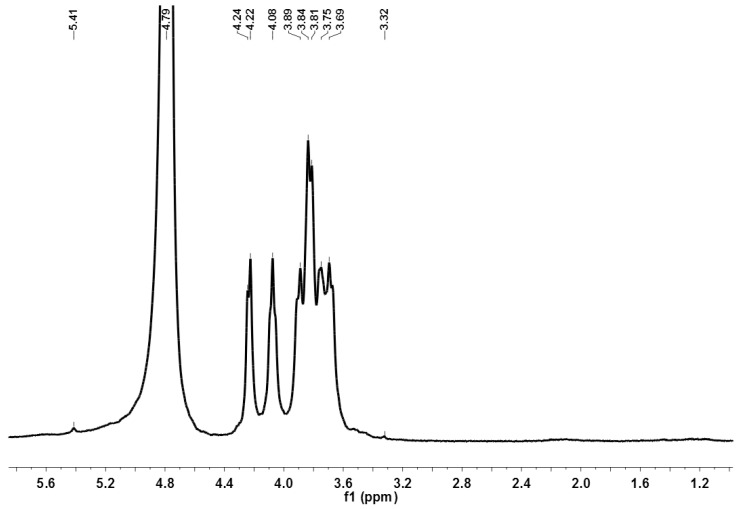
^1^H-NMR spectrum of DLHP.

FT-IR spectra of DLHP showed the characteristic absorptive peaks of glycosidic structures (Figure 3). The strong and broad absorptive peak at 3414.06 cm^−1^ was caused by the stretch vibration of O–H bond. The signal at 2922.12 cm^−1^ was associated with the C–H stretch vibration, while the absorption at 1385 cm^−1^ represented the C–H bending vibration. The band at 1632.88 cm^−1^ could be attributed to the stretching vibration of a free carboxyl group [15]. The strong absorption in the region of 1000–1200 cm^−1^ was due to the C–O stretching vibration of pyranglycoside linkage. Moreover, the obvious absorption band at 817.76 cm^−1^ revealed the polysaccharide containing α-type glycosidic bond in structure [16]. By ^1^H-NMR spectroscopy, the preponderant signals between 3.2 and 4.3 ppm were assigned to the protons of a glycosidic ring (Figure 4). The anomeric proton signals were found at about δ 5.41 ppm, suggesting the existence of α-glycosidic bonds, consistent with IR data. For LHP, the similar characteristic bands/signals were also found in both spectra.

### 2.3. Antioxidant Activities

#### 2.3.1. 1,1-Diphenyl-2-picrylhydrazyl (DPPH) Radical Scavenging Activity

**Figure 5:**
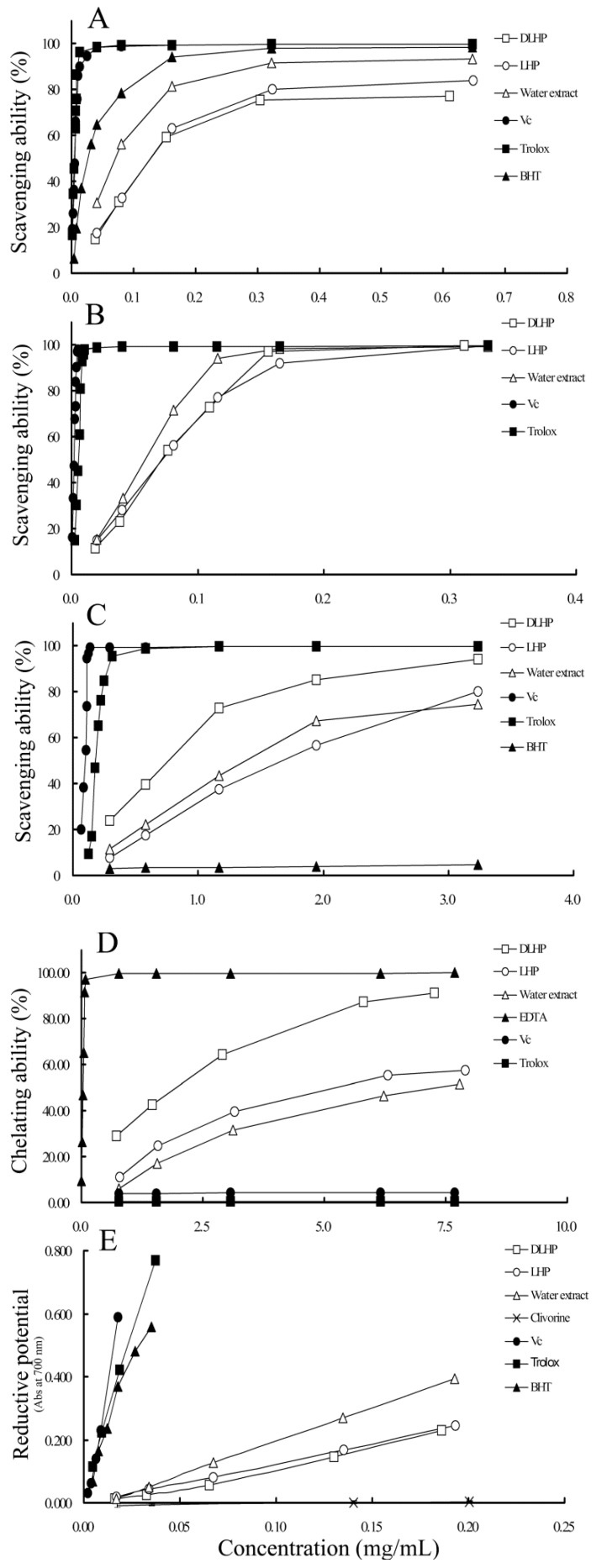
Scavenging effects on 1,1-diphenyl-2-picrylhydrazyl (DPPH) radical (**A**); 2,2′-azinobis (3-ethylbenzothiazoline-6-sulfonic acid) (ABTS) radical (**B**); and hydroxyl radical (**C**); metal chelating activity (**D**); and reductive potential (**E**) of DLHP, LHP and water extract.

As a stable free radical, 1,1-diphenyl-2-picrylhydrazyl (DPPH) is widely used to test free-radical scavenging activity of natural antioxidants [17]. Some antioxidants, e.g., polysaccharides, can neutralize the DPPH free radicals and terminate their chain reaction by direct electron-transferring [18]. The extent of decolorization from purple to yellow depends on the scavenging potential and concentrations of the samples. As shown in Table 3, the IC_50_ value of DLHP against DPPH^•^ was 142.3 ± 2.0 μg/mL. In the range 38.1–610.2 μg/mL, its inhibition percentage increased from 14.9% to 77.1%, showing concentration dependence (Figure 5A). In comparison, the scavenging effect of DLHP (75.4% ± 0.4%) at 305.1 μg/mL was compatible to those of V_C_ (76.0% ± 0.7%) at 8.66 μg/mL, butylated hydroxytoluene (BHT) (75.2% ± 0.5%) at 62.4 μg/mL, and Trolox (75.9% ± 0.4%) at 7.60 μg/mL. This result is comparable to or even better than those by other plant-derived polysaccharides reported, for instant, two polysaccharides isolated from *Elaeagnus angustifolia*, showing IC_50_ values of 0.27 and 0.38 mg/mL, respectively [19]; the polysaccharide from *Ligusticum chuanxiong* rhizomes, showing the scavenging rate 66.5% at 375 μg/mL [18]; and the sulfated *Codonopsis pilosula* (*C. pilosula*) polysaccharide with enhanced antioxidative and hepatoprotective activities, showing about 80% at 1.25 mg/mL [9]. Moreover, LHP also exhibited high scavenging activity with IC_50_ value of 127.1 ± 2.4 μg/mL. These results indicate that the LH polysaccharides possess significant DPPH^•^ scavenging capacity.

It is worth noting that LHP and water extract exhibited higher scavenging activities than DLHP while their total carbohydrate contents (65.2% ± 0.5% for LHP and 12.0% ± 0.5% for water extract) were lower than that of DLHP. This is probably due to their higher contents of total polyphenols (Table 2). Phenolics are believed to be one of the most effective free radical scavengers with high potential to donate electrons and/or hydrogen atoms to the free radicals [17]. As regards, both polysaccharides and phenolics might contribute to the DPPH^•^ scavenging activity of the water extract.

#### 2.3.2. 2,2′-Azinobis (3-ethylbenzothiazoline-6-sulfonic acid) (ABTS) Radical Scavenging Activity

ABTS (2,2′-azinobis (3-ethylbenzothiazoline-6-sulfonic acid)) assay is another commonly-used method to evaluate the scavenging capacities of antioxidants. Similar to DPPH, antioxidants can neutralize ABTS radical cation (ABTS^•+^) by direct electron transfer mechanism [17]. This method can be applied to either hydrophilic or lipophilic substances. Figure 5B showed that the ABTS^•+^ scavenging activity of DLHP was the concentration dependent within the range 19.1–311.6 μg/mL, of which the IC_50_ value was measured to be 62.4 ± 0.3 μg/mL (Table 3). At 155.8 μg/mL, the scavenging rate was 97.2% ± 0.6%, better than 66.2% of the best *C. pilosula* polysaccharides at 238.1 μg/mL [9]. Comparatively, LHP showed almost same scavenging potential as DLHP, while the water extract was the most powerful one with the IC_50_ value of 48.5 ± 0.3 μg/mL. Similar to DPPH, high content of total polyphenols may contribute to the activity of water extract.

#### 2.3.3. Hydroxyl Radical Scavenging Activity

Hydroxyl radicals are highly reactive towards proteins, lipids and DNA, and severely harmful for cell survival when overproduced [5]. Removal of the radicals is thus important for the living systems to maintain the redox homeostasis. As shown in Figure 5C, the scavenging activity of DLHP, LHP and the water extract increased with increase in the concentration range 0.2–3.2 mg/mL. The scavenging effect of DLHP was about 95% at 3.05 mg/mL and 73% at 1.10 mg/mL. This value is better than those of *C. pilosula* polysaccharides, which scavenging percentages to hydroxyl radical were found to be approximately 36%–46% at 0.91 mg/mL [9]. According to the IC_50_ values, the scavenging activity of DLHP was significantly higher than those of LHP and water extract (Table 3). As for the water extract, its maximum scavenging rate was lower than that of LHP even with a smaller IC_50_ value (Figure 5C, Table 3). Its activity may be due to the existence and synergism of many active or even toxic components besides polysaccharides, for instant, clivorine, as a representative toxic PA component, also showed some activity in this assay (Table 3).

#### 2.3.4. Ferrous Metal Ions Chelating Activity

Some redox-active transition metals such as iron (Fe) are essential elements required for the growth and survival of mammals, whereas they also play the catalytic role in the free radical formation and cause various oxidative damages [20]. The above-mentioned Fenton reaction is just involved by ferrous iron (Fe^2+^)-dependent decomposition of hydrogen peroxide to generate hydroxyl radicals. Accordingly, the Fe^2+^ chelating activity is also an important attribute of the antioxidants. As shown in Figure 5D, the chelating effects of all samples were ascended with the increase of concentrations (0.73–7.89 mg/mL). At 7.27 mg/mL, the chelating effect of DLHP could reach 91.2% ± 0.54%. This value was comparable to that of EDTA (91.8% ± 0.29%) at 0.06 mg/mL and remarkably higher than those of V_C_ and Trolox. V_C_ was supposed to undergo degradation amid the presence of FeCl_2_ in the system, which physiological form, ascorbate anion, can also reduce Fe(III) to Fe(II) [20].

Based on the IC_50_ values, the ranking order of the activity was DLHP > LHP > water extract, which corresponded to their polysaccharide contents (Table 2 and Table 3). This result revealed important role of polysaccharide components. It has been reported that the presence of protein may cause a decrease of metal ion chelating activity while some Fe^2+^ chelating groups (e.g., –COOH) in structure may benefit the chelating effects of polysaccharides [21,22]. Therefore, the high chelating ability of DLHP may partly depend on the relatively high percentage of uronic acids in structure and much less protein impurities (Table 2). Compared with other herb-derived polysaccharides, the LH polysaccharides also showed a greater potential, for instant, the Fe^2+^ chelating effect of the most powerful polysaccharide fraction from *Glycyrrhizae Radix* showed chelating ability close to 40% at 4 mg/mL [23], just comparable to that of DLHP at 1.5 mg/mL.

#### 2.3.5. Reducing Power

The antioxidant power of polysaccharides is usually evaluated by the FRP assay in water solution [21]. Figure 5E showed that the reductive potentials of DLHP, LHP and water extract increased proportionally with the increase of concentrations, with absorbance values of 0.23, 0.24 and 0.40, respectively, when the concentration was about 0.19 mg/mL. Clivorine showed no reducing power (almost no absorbance) up to 0.2 mg/mL. In comparison, the equivalent reducing power (about 0.23) of V_C_ and Trolox were achieved at 0.009 mg/mL; this concentration was high enough for them to clear more than 75% of DPPH^•^ and 95% of ABTS^•+^ (Figure 5A,B). Given the good performance in electron-transfer based DPPH/ABTS assays, the LH polysaccharides could be the effective electron donors for the reduction of Fe^3+^/ferricyanide.

Similar to DPPH assay, the ranking order of reducing power was also water extract > LHP > DLHP. Because the activity may be affected by the molecular weight (*M*_W_) of polysaccharides, that is, the lower *M*_W_, the more the exposed reducing end and the stronger the reducing power [21]; the lower reducing power of DLHP than LHP may be attributed to an increase of *M*_W_ distribution after dialysis (Table 2). Furthermore, a lower activity of DLHP may be due to removal of some high reducing components (e.g., phenolics) (Table 2) [17]. In addition, a strict deproteination may also cause a decline in reducing power [21]. Therefore, the elevated activities by LHP and water extract may be partly ascribed to the presence of these active impurities.

### 2.4. In Vitro Anti-Hyperglycemic Study

#### 2.4.1. α-Glucosidase Inhibitory Activity

**Figure 6:**
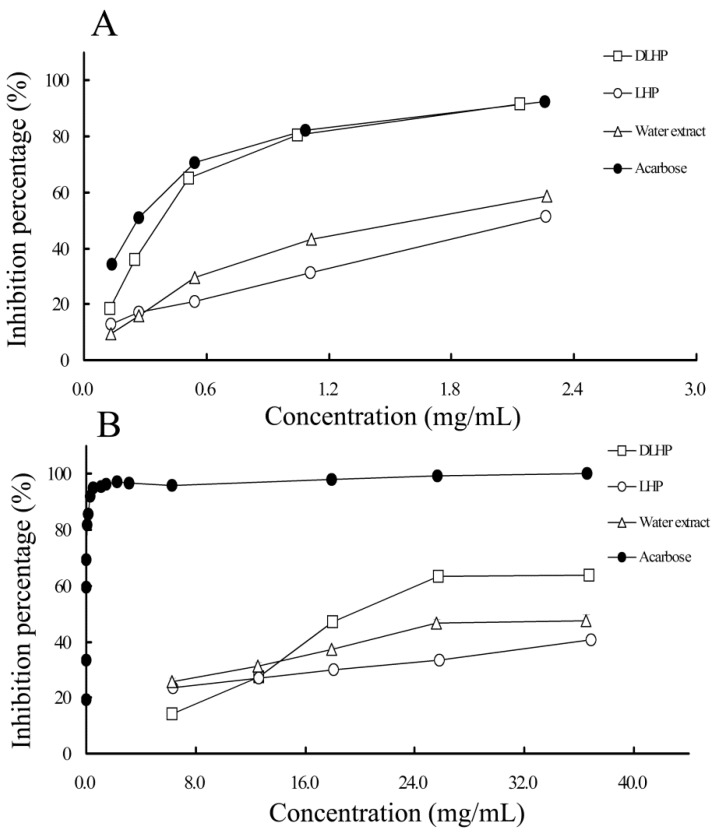
The inhibitory activities of DLHP, LHP and water extract on: α-glucosidase (**A**); and α-amylase (**B**).

As a typical characteristic of diabetes, chronic hyperglycemia is strongly implicated in the pathogenesis of diabetic complications [6]. One of the therapeutic approaches is to retard absorption of glucose into the bloodstream by inhibition of carbohydrate-hydrolyzing enzymes, such as α-glucosidase and α-amylase in the small intestine [24]. Both enzymes can digest dietary starch into glucose, while α-glucosidase is the key enzyme finalizing the catalyzing process. Many plant-derived polysaccharides have been reported to have anti-hyperglycemic potential through the α-glucosidase inhibition [25,26]. In this assay, a series of concentrations (0.1–2.2 mg/mL) were tested for each sample to determine the percentage inhibition of α-glucosidase. As shown in Figure 6A, all samples exhibited the inhibitory effect in a concentration-dependent manner. The α-glucosidase inhibitory activity (IC_50_) was found to be 242.0 ± 3.7 and 358.4 ± 0.6 μg/mL for acarbose and DLHP, respectively. In terms of average 50% inhibition, DLHP has almost 77% equivalent potency to acarbose; while in the concentration higher than 0.5 mg/mL, the activity of DLHP was almost equal to that of acarbose (Figure 6A). Acarbose is an important α-glucosidase inhibitor used for the treatment of Type 2 diabetes [8]. This result indicated that DLHP was a potential α-glucosidase inhibitor.

In comparison, DLHP’s IC_50_ was significantly lower than those of LHP and water extract, while water extract revealed more powerful activity against α-glucosidase than LHP (Table 3). Besides the difference in polysaccharide content, the structural features (e.g., *M*_W_) may also be important towards enzyme inhibition [8]. As such, the higher activity of DLHP than LHP may be ascribed to its larger *M*_W_ and higher uronic acids content (Table 2). On the other hand, the relatively higher activity of water extract than LHP may partly rely on its larger content of polyphenols or other bioactive compounds with the inhibitory potentials [27].

#### 2.4.2. α-Amylase Inhibitory Activity

α-Amylase is another target enzyme for the control of hyperglycemia [24]. Figure 6B showed that the inhibitory effects of DLHP, LHP and water extract were significantly lower than the positive control acarbose even though they increased in a concentration-dependent manner. The IC_50_ value of DLHP was 21.2 ± 0.3 mg/mL while that of acarbose was 15.5 ± 0.3 μg/mL (Table 3). This result was incompatible with that obtained in the α-glucosidase inhibitory assay (Figure 6A). It could be deduced that almost 60 times more DLHP is needed to inhibit α-amylase to the same degree as α-glucosidase (0.358 mg/mL). This difference in inhibition, especially a relatively high α-glucosidase inhibition and mild α-amylase inhibition is preferred because an excessive inhibition of α-amylase may lead to side effects caused by the abnormal bacterial fermentation of undigested carbohydrates in the colon [8]. The difference may depend on the structural features (e.g., *M*_W_, uronic acids content, and conformation) of DLHP, which variation led to the different mode of action between α-glucosidase and α-amylase [26].

In comparison to DLHP, the 50% enzyme inhibition by LHP and water extract could not be achieved even at the maximum test concentrations. But their performances in the activity were different at different concentrations; the ranking order changed at the concentrations of less than 12 mg/mL (Figure 6B). This postulates that larger amount of small components in LHP and water extract, such as polyphenols and PAs, may be involved in the inhibition. A further study showed that clivorine had an IC_50_ value of 6.67 mg/mL, which means relatively higher inhibition activity than DLHP. The variety and amount of these active compounds in LHP and water extract may cause definitely different effects.

Many studies suggest that the bioactivity of polysaccharide is closely associated with its structure [7,8,26]. The *M*_W_, monosaccharide composition, the configuration of glycosidic bonds, and the substituents (e.g., –COOH) of the polysaccharides as well as the spatial structure may all affect the bioactivity. Moreover, the bioactivity may not be the result of any single structural factor but of many ones when combined in an appropriate pattern [15,28]. As shown above, the purification of LHP led not only to the increased polysaccharide content of DLHP but also the larger *M*_W_ distribution, higher content of uronic acids and varied molar ratio of monosaccharide composition, which exert the differential influence on the antioxidant properties such as hydroxyl radical scavenging activity and reducing power. The structural differences may also result in different anti-hyperglycemic potential of DLHP and LHP or even differential mode of inhibition of α-glucosidase and α-amylase (Table 3). However, due to the lack of detailed structural information and *in vivo* activity results, a comprehensive understanding of these effects and underlying mechanisms is far from complete, and further studies are warranted.

## 3. Materials and Methods

### 3.1. Materials and Reagents

The roots and rhizomes of *Ligularia hodgsonii* (*L. hodgsonii*) were collected from *E’mei* Mountain of Sichuan Province, China, and authenticated based on the morphologic appearance, microscopic and physiochemical analyses [1]. Voucher specimens (LH1701/3) were deposited in School of Pharmaceutical Sciences, Wuhan University. The dried herbal materials were ground in a blender to obtain a fine powder (particle size: 0.25 μm) before use. Clivorine was isolated from the ethanolic extract of *L. hodgsonii* with the purity over 98% by MS, NMR and HPLC analyses [11]. DPPH, ABTS, 4-nitrophenyl-α-d-glucopyranoside (pNPG), α-glucosidase from *Saccharomyces cerevisiae*, and α-amylase from porcine pancreas were purchased from Sigma-Aldrich (St. Louis, MO, USA). 1-phenyl-3-methyl-5-pyrazolone (PMP), ascorbic acid (V_C_), 6-hydroxy-2,5,7,8-tetramethylchroman-2-carboxylic acid (Trolox), butylated hydroxytoluene (BHT), gallic acid, mannose, rhamnose, glucuronic acid, galacturonic acid, glucose, galactose and arabinose were obtained from Aladdin Industrial Corporation (Shanghai, China, the purities ≥ 98%). All other chemicals used were of analytical grade and purchased from Sinopharm Chemical Reagent Co., Ltd. (Shanghai, China).

### 3.2. Extraction of Crude Polysaccharides

The water extract was obtained by refluxing 500 g of powdered herbal materials with distilled water as previously described [1]. For the extraction of polysaccharides, the powders were firstly defatted with 95% ethanol at 60 °C for 6 times in a reflux apparatus, and then dried to a constant weight. Each dried and pretreated residual (2 g) was then extracted with de-ionized water at the following designated conditions. After extraction, some routine processes were fine-tuned for precipitation: the supernatants were collected, concentrated (1 mL equal to 0.25 g herbal material) and precipitated by the addition of 5.3-fold volume of 95% ethanol to a final concentration of 80% (*v*/*v*) [21]. The mixture was stirred vigorously and then kept overnight at 4 °C. The precipitates were separated by centrifugation at 7669× *g* for 10 min; the product was washed repeatedly by absolute ethanol and diethyl ether, and dried by vacuum to obtain the crude polysaccharides (LHP). The total carbohydrate content was determined by the phenol-sulfuric acid method using glucose as standard [29]. The polysaccharide yield (%) was calculated as follows: polysaccharide yield (%, *w*/*w*) = (weight of total polysaccharides (g)/weight of dried herbal powder (g)) × 100.

### 3.3. Single Factor Experiments

Single factor experiments were used for obtaining the preliminary range of the extraction variables, namely extraction temperature (65, 75, 85, and 95 °C), extraction time (1, 1.5, 2, 3, and 4 h), W/M ratio (6, 12, 18, 24, 30, and 36 mL/g), and extraction frequency (1, 2, and 3 times). Only one variable could be changed in each experiment while the others were kept constant. The polysaccharide yield of LHP was the response variable.

### 3.4. Orthogonal Array Test Design

On the basis of single factor experiments, an orthogonal *L*_9_ (3^4^) test was designed to optimize extraction condition of the LH polysaccharides. As shown in Table 1, the experiments were carried out with four factors and three levels, namely A (temperature, 80, 85, and 90 °C), B (time, 2, 3, and 4 h), C (W/M ratio, 24, 30, and 36 mL/g) and D (frequency, 1, 2, and 3 times). The different conditions were evaluated by *Z*-comprehensive score method [30], in which there are one positive attribute, the polysaccharide yield, and three negative attributes, that is, proteins, total polyphenols and clivorine. Each attribute was assigned the different weighting coefficient, for instance, proteins and total polyphenols were −0.2 each, and clivorine was −0.6, respectively, according to their impacts on the extraction quality of polysaccharides. The composite scores plus 5 was computed to avoid negative results. The calculation formula was as follows:

(1) $$Z_{i}=(X_{i}-\bar{X_{i}})/S_{i}$$

*Z*-Comprehensive score (*Z_ij_*) = ∑ *Z_i_*W*_j_* + 5 = *Z*_1_ + *Z*_2_ × (−0.2) + *Z*_3_ × (−0.2) + *Z*_4_ × (−0.6) + 5 (2)

where $\bar{X_{i}}$ is the average measured value, *X_i_* the actual measured value, *S_i_* the standard deviation, W*_j_* is the weight, *Z*_1_ the polysaccharide yield (%, *w*/*w*), *Z*_2_ protein content (%, *w*/*w*), *Z*_3_ total polyphenol content (%, *w*/*w*), and *Z*_4_ the clivorine content (%, *w*/*w*).

The optimized condition was further verified by the additional experiments. Three powdered herbal samples (each about 2 g) were extracted under the optimal process and the *Z*-scores calculated as above.

### 3.5. Removal of Impurities in Crude Polysaccharides

LHP was dissolved again in water, and extracted by the Sevag method to exclude the dissociative protein [31]. Then the solution was precipitated by ethanol again, after that the sediment was dissolved in water (10 mg/mL) for dialysis. The impurities such as salts and molecules with molecular weight (*M*_W_) less than 12,000 Da could be removed by the dialysis. Prior to dialysis, the membrane (molecular weight cut off 12,000 Da) was cut into a suitable size and boiled in de-ionized water for 15 min. Dialysis was proceeded for 6 days, during which the solution was stirred and replaced with fresh distilled water frequently until the process was complete. Dialysis solution was mixed with 5.3-fold volume of 95% ethanol. The mixture was further treated as described in Section 3.2, affording the dialyzed polysaccharide (DLHP).

### 3.6. Preliminary Characterization of the Polysaccharides

#### 3.6.1. General Methods

The purity of polysaccharides in terms of starch residual and carbohydrate identity were examined by iodine-potassium iodide and Molish reagents [28]. Total carbohydrate content was determined by the above-mentioned phenol-sulfuric acid method [29]. Protein was measured by the Bradford’s method using bovine serum albumin as the standard [32]. Total polyphenol content was determined using the Folin-Ciocalteu assay, with Gallic acid as the standard [33]. The clivorine content was estimated by a validated method in our laboratory [11,13]. The limit of quantification and limit of detection were determined to be 0.153 and 0.046 μg/mL, respectively. Uronic acid content was determined by photometry with *m*-hydroxybiphenyl reagent using d-glucuronic acid as the standard [34]. Sulfate radical content was determined by the barium chloride-gelatin method using K_2_SO_4_ as a standard [35].

#### 3.6.2. Analysis of Monosaccharide Composition

For monosaccharide composition analysis, the polysaccharide powder (20 mg) was hydrolyzed with 3 mL of 2 M trifluoroacetic acid (110 °C, 6 h). The hydrolysate was co-evaporated with methanol repeatedly to dryness and then converted to its 1-phenyl-3-methyl-5-pyrazolone (PMP) derivative according to the method of Lv *et al.*, 2009 [36]. The PMP derivatives of monosaccharide standards were prepared in the same way. Each sample solution was filtered through a 0.45 μm membrane and analyzed by a reported HPLC method [36] with slight modification: wavelength for UV detection at 245 nm, the mobile phase consisting of acetonitrile and 0.045% KH_2_PO_4_-0.025% triethylamine buffer (pH 7.0), eluted with a linear gradient of 17% to 20% acetonitrile from 2 to 20 min.

#### 3.6.3. Determination of Molecular Weight

The weight-average molecular weight (*M*_W_) of the polysaccharides was estimated by size exclusion chromatography (SEC) using a method of Xu *et al.* [37]. An instrumental system contained a pump (515 HPLC, Waters, Milford, MA, USA), a gel-filtration chromatographic column of TSK-GEL GMPWxL column (7.8 mm × 300 mm, Tokyo, Japan), a multiangle laser light scattering (MALLS) instrument and a differential refractive index (RI) detector (Wyatt Technology, Santa Barbara, CA, USA). The measurements of polysaccharides *M*_W_ were achieved according to light-scattering intensities of 18 angles from 14° to 163° at 25 °C. The Astra software (Version 6.1.1) (Wyatt Technology) was utilized for data acquisition and analysis. The specific RI increment (d*n*/d*c*) was set at 0.14 mL/g in aqueous solution. The mobile phase was 0.1 M NaNO_3_ (pH 7.0) solution filtrated on 0.22 μm filters, at a flow rate of 0.5 mL/min. The polysaccharide samples (5 mg) were dissolved in 5 mL of 0.1 M NaNO_3_ solution and centrifuged at 19,070× *g* for 10 min, and then passed through a 0.45 μm filter.

#### 3.6.4. Infrared (IR) Analysis

The dried polysaccharide (5 mg) was mixed with KBr powder, ground and pressed into a 1 mm thick disk for analysis. The IR spectra of the LH polysaccharides were measured on a Nicolet Nexus 470 FT-IR spectrometer (Waltham, MA, USA) in the frequency range of 4000–400 cm^−1^.

#### 3.6.5. ^1^H Nuclear Magnetic Resonance (^1^H NMR)

The sample was dissolved in D_2_O to be the concentration of 10 mg/mL. The NMR spectra were recorded on a Bruker AV400 spectrometer (Bruker, Gotthardstrasse, Switzerland) operating at 400 MHz for ^1^H.

### 3.7. In Vitro Antioxidant Activity Assays

#### 3.7.1. DPPH Radical Scavenging Assay

The DPPH quenching ability was measured by the reported procedure with a minor modification [1]. Briefly, 0.3 mL of the aqueous solution of polysaccharides was added to 2.7 mL of 0.07 mM DPPH^•^ methanolic solution; the mixture was shaken vigorously and allowed to stand for 1 h before the absorbance was measured at 516 nm. V_C_, BHT and Trolox were used as the positive controls. The scavenging effect was calculated by the following Equation (3):

(3) $$\text{Scavenging effect (\%)}\;=\frac{\left[A_{0}-(A_{1}-A_{2})\right]}{A_{0}}\times 100$$

where *A*_0_ was the absorbance of the control samples (water instead of tested sample), *A*_1_ was the absorbance of the test samples and *A*_2_ was the absorbance of the samples only (without free radicals).

#### 3.7.2. ABTS Radical Scavenging Assay

The activity was also measured by the published method [1]. Briefly, the ABTS^•+^ was generated by dissolving ABTS diammonium salt in 2.45 mM potassium persulfate to be a concentration of 7 mM, and then keep in dark at room temperature for 16 h. Before the measurement, the ABTS^•+^ solution was diluted with the distilled water to an absorbance of 0.70 ± 0.02 at 734 nm. The diluted solution (2.7 mL) was added to 0.3 mL sample solution. The reaction mixture was then measured at 734 nm after incubation in dark for 30 min at 30 °C. Similarly, V_C_, BHT and Trolox were used as the positive controls. The scavenging activity of test samples was calculated by the same equation as Equation (3) except that the ABTS^•+^ solution was used for measurements.

#### 3.7.3. Hydroxyl Radical Scavenging Assay

The assay was investigated as previously described using a well-known Fenton reaction with minor modification [14]. Briefly, 1.0 mL of different sample solutions (in distilled water) were mixed successively with 1.0 mL salicylic acid-ethanol solution (6 mM), 1.0 mL FeSO_4_ (2 mM), and 1.0 mL H_2_O_2_ (6 mM). The reaction solution was incubated at 37 °C for 30 min. The absorbance of the mixtures was measured at 510 nm, using distilled water as blank. V_C_ and Trolox were used as the positive controls. The scavenging activity was calculated using the following Equation (4):

(4) $$\text{Scavenging effect (\%)}\;=\frac{\left[A_{0}-(A_{1}-A_{2})\right]}{A_{0}}\times 100$$

where *A*_0_ was the absorbance of the control samples, *A*_1_ was the absorbance of the test samples and *A*_2_ was the absorbance of the test samples without H_2_O_2_.

#### 3.7.4. Ferrous Metal Ions Chelating Activity

The ferrous ion-chelating ability was determined as described by Lin *et al.* [21]. Briefly, different concentrations of the test samples were prepared in de-ionized water, 0.1 mL of sample was mixed with 0.01 mL of FeCl_2_ (2 mM), 0.02 mL of ferrozine (5 mM) and 0.27 mL of distilled water. After incubating at room temperature for 10 min, the absorbance was measured at 562 nm, where the lower the absorbance, the higher the chelating ability. EDTA, V_C_ and Trolox were used as standards for comparison. The chelating ability was calculated using the following Equation (5):

(5) $$\text{Chelating ability (\%)}\;=\frac{\left[A_{0}-(A_{1}-A_{2})\right]}{A_{0}}\times 100$$

where *A*_0_ was the absorbance of the control samples, *A*_1_ was the absorbance of the test samples and *A*_2_ was the absorbance of the test samples without ferrozine.

#### 3.7.5. Ferric Reducing Power (FRP)

The reducing power was determined as also in the reference [21] with minor changes. Each polysaccharide solution (0.2 mL) was mixed with 2.0 mL of 0.2 M sodium phosphate buffer (pH 6.6) and 2.0 mL of 1% potassium ferricyanide, and the mixture was incubated at 50 °C for 20 min. The reaction was terminated by the addition of 2.0 mL of 10% trichloroacetic acid, and then the mixture was centrifuged at 955× *g* for 10 min. The supernatant (2 mL) was mixed with 2 mL of de-ionized water and 0.4 mL of 0.1% ferric chloride, and the absorbance was measured at 700 nm against blank. Increased absorbance indicates an increase of reducing power. V_C_, BHT and Trolox were used as positive controls.

### 3.8. In Vitro Anti-Hyperglycemic Studies

#### 3.8.1. α-Glucosidase Inhibitory Activity

The α-glucosidase inhibitory activity was determined using the method described by Kim *et al.* [8] with slight modification. Briefly, sample solution (50 μL, prepared in buffer), α-glucosidase (50 μL, 1 U/mL) and 0.1 M phosphate buffer (900 μL, pH 6.8) were mixed and maintained at 37 °C for 10 min. Then, 100 μL of 5 mM pNPG was added and incubated at 37 °C for another 30 min. The reaction was terminated by adding 4 mL of 0.1 M NaCO_3_ solution. The absorbance at 400 nm was measured, using buffer as zero-setting solution. Acarbose was used as positive control. The inhibitory rate was calculated by the following Equation (6):

(6) $$\text{Inhibition activity (\%)}\;=\frac{\left[A_{0}-(A_{1}-A_{2})\right]}{A_{0}}\times 100$$

where *A*_0_ was the absorbance of the control samples, *A*_1_ was the absorbance of the test samples and *A*_2_ was the absorbance of the test samples without enzymes.

#### 3.8.2. α-Amylase Inhibitory Activity

The α-amylase activity was measured according to the reference [8]. A mixture of 20 mM phosphate buffer (100 μL, pH 6.9, with 0.0067 M sodium chloride), sample solution (100 μL) and α-amylase solution (100 μL, 0.5 mg/mL) was pre-incubated at 37 °C for 10 min. Then, 200 μL of 1% starch solution in the pH 6.9 buffer was added and incubated at 37 °C for another 10 min. The reaction was stopped with 200 μL of the DNS reagent (1% 3,5-dinitrosalicylic acid and 12% sodium potassium tartrate in 0.4 M NaOH) and tubes were incubated at 100 °C for 10 min. After cooling in ice-water, the reaction mixture was diluted by adding 4 mL of distilled water and absorbance was measured at 540 nm. Acarbose was also used as positive control. Inhibitory activity was calculated by the same equation as Equation (6), except that the absorbance was recorded as stated above.

### 3.9. Statistical Analyses

Data of all experiments were expressed as means ± standard deviation (SD) of three replicates and evaluated by one way analysis of variance (ANOVA) followed by Student’s *t*-test. *p* < 0.05 was considered to be statistically significant. In above assays, the IC_50_ values were defined as the concentrations required for scavenging 50% of free radicals (antioxidant assay) or inhibiting 50% of the hydrolytic enzyme activity (anti-hyperglycemic study) and calculated by probit analyses.

## 4. Conclusions

In this study, optimization extraction, preliminary characterization, antioxidant and anti-hyperglycemic activities *in vitro* of the LH polysaccharides were carried out. The optimum extraction conditions of LHP were determined using single-factor experiments and orthogonal array test. DLHP was purified from LHP via Sevag deproteinization and dialysis. Most of the impurities especially toxic PA components were removed. Through the physicochemical analyses, both DLHP and LHP showed a major polysaccharide component with the similar monosaccharide composition but different *M*_W_, uronic acid content, and molar ratio of the monosaccharide composition. DLHP and LHP diverge significantly in both antioxidant and anti-hyperglycemic assays, which may be partly due to the structural differences. DLHP displayed significant scavenging activities against DPPH, ABTS and hydroxyl radicals; ferrous ions chelating capacity; and high inhibitory activities against α-glucosidase, most of which were better than LHP. Moreover, DLHP showed a weak inhibition of α-amylase, suggesting its selectivity in anti-hyperglycemic activity. In a view of water extract, the chemical complexity was prominent; its activities may be the combined effects of many active components including polysaccharides, polyphenols and even toxic PA components. These findings are important in the development of DLHP towards not only a new and safe antioxidant and hypoglycemic agent but possible protective agent against PA-induced oxidative damage also. However, the detailed structural characterization, functions and mechanisms of action will be further elucidated.

## Figures and Tables

**Table 1:** Factors and levels for *L*_9_ (3^4^) orthogonal array test and the experimental results.

No.	A (°C)	B (h)	C (mL/g)	D (Times)	Polysaccharide Yield/%	Proteins/%	Total Polyphenols/%	Clivorine/(×10^−4^) %	*Z*-Comprehensive Scoring
1	1 (80)	1 (2)	1 (24)	1 (1)	21.5 ± 1.1	2.15 ± 0.06	1.73 ± 0.04	2.29 ± 0.04	2.78 ± 0.57
2	1	2 (3)	2 (30)	2 (2)	27.7 ± 0.7	1.94 ± 0.01	1.73 ± 0.03	1.87 ± 0.03	5.29 ± 0.23
3	1	3 (4)	3 (36)	3 (3)	26.9 ± 1.9	2.50 ± 0.11	1.86 ± 0.02	1.99 ± 0.02	3.93 ± 0.56
4	2 (85)	1	2	3	32.1 ± 2.2	2.16 ± 0.09	1.87 ± 0.03	2.11 ± 0.04	5.57 ± 0.45
5	2	2	3	1	27.6 ± 2.4	2.22 ± 0.10	1.69 ± 0.02	0.83 ± 0.01	6.35 ± 0.76
6	2	3	1	2	29.1 ± 1.4	2.22 ± 0.06	1.79 ± 0.03	1.27 ± 0.01	5.87 ± 0.47
7	3 (90)	1	3	2	28.7 ± 1.0	2.01 ± 0.06	1.81 ± 0.01	1.40 ± 0.01	5.78 ± 0.29
8	3	2	1	3	28.1 ± 1.3	2.46 ± 0.06	1.87 ± 0.04	1.80 ± 0.02	4.58 ± 0.39
9	3	3	2	1	22.7 ± 2.7	1.91 ± 0.07	1.77 ± 0.03	0.91 ± 0.01	4.87 ± 0.61
k_1_ *^a^*	3.99	4.74	4.40	4.57	-	-	-	-	-
k_2_ *^a^*	5.98	5.37	5.25	5.68	-	-	-	-	-
k_3_ *^a^*	5.04	4.89	5.35	4.75	-	-	-	-	-
*R ^b^*	1.99	0.62	0.96	1.12	-	-	-	-	-

*^a^* Average *Z*-score of each level about the extraction efficiency of *Ligularia hodgsonii* polysaccharides (LHP); *^b^* *R* value means range between three average *Z*-scores of each level.

**Table 2:** Physicochemical properties of the *Ligularia hodgsonii* polysaccharides and water extract.

Indices	DLHP	LHP	Water Extract
Total carbohydrates (%)	86.9 ± 0.4	65.2 ± 0.5	12.0 ± 0.5
Protein (%)	1.04 ± 0.02	2.25 ± 0.04	1.53 ± 0.03
Total polyphenols (%)	0.513 ± 0.008	1.68 ± 0.01	22.7 ± 0.3
Clivorine (%)	ND *^c^*	0.819 ± 0.009 (×10^−4^)	0.394 ± 0.008 *^e^*
Uronic acids (%)	5.03 ± 0.08	4.05 ± 0.08	-
Sulfuric radical (%)	ND *^c^*	ND *^c^*	-
Molecular weight (×10^5^ Da)	1.17 ± 0.03	0.11 ± 0.01	-
*M*_W_/*M*_n_ *^d^*	1.42 (±4.51%)	1.15 (±11.8%)	-
Mass Fraction/%	91.6 ± 0.8	99.3 ± 0.4	-

*^c^* Not detected; *^d^* polydispersity index; *^e^* previously reported by Tang *et al.* [11]; -, not determined; DLHP, dialyzed LHP.

**Table 3:** The IC_50_ (μg/mL) values of the *Ligularia hodgsonii* polysaccharides, water extract and positive controls on antioxidant and anti-hyperglycemic activities.

Samples	DPPH^•^ Scavenging	ABTS^•+^ Scavenging	^•^OH Scavenging	Fe^2+^ Chelating	Inhibition of α-Glucosidase	Inhibition of α-Amylase
DLHP	142.3 ± 2.0	62.4 ± 0.3	621.7 ± 6.7	1511.3 ± 7.6	358.4 ± 0.6	(21.2 ± 0.3) × 10^3^
LHP	127.1 ± 2.4	61.4 ± 0.7	1530.7 ± 14.3	5181.3 ± 78.1	2491.7 ± 132.8	(114.8 ± 3.6) × 10^3^ *^f^*
Water extract	62.3 ± 0.8	48.5 ± 0.3	1346.0 ± 13.2	8735.3 ± 325.7	1604.1 ± 3.9	(38.4 ± 3.4) × 10^3^ *^f^*
Clivorine	/	/	435.7 ± 3.8	/	/	(6.67 ± 0.03) × 10^3^
V_C_	4.71 ± 0.02	1.66 ± 0.01	87.3 ± 0.1	/	-	-
BHT	25.1 ± 0.1	-	/	-	-	-
Trolox	4.15 ± 0.02	4.94 ± 0.05	183.3 ± 1.6	/	-	-
EDTA	-	-	-	28.7 ± 0.2	-	-
Acarbose	-	-	-	-	242.0 ± 3.7	15.5 ± 0.3

*^f^* Estimated value; “/” no IC_50_ value obtained; -, not determined. There are significant differences (*p* < 0.05) between DLHP, LHP and water extract in all assays but DLHP and LHP in ABTS^•+^ assay. BHT, butylated hydroxytoluene.
